# Electrical impedance tomography to determine optimal positive end-expiratory pressure in severe chronic obstructive pulmonary disease

**DOI:** 10.1186/s13054-016-1475-2

**Published:** 2016-09-22

**Authors:** Eirini Kostakou, Nicholas Barrett, Luigi Camporota

**Affiliations:** Department of Adult Critical Care, Guy’s and St Thomas’ NHS Foundation Trust, King’s Health Partners, St Thomas’ Hospital, 1st Floor East Wing, Westminster Bridge Road, London, SE1 7EH UK

**Keywords:** Chronic obstructive pulmonary disease, Electrical impedance tomography

Dynamic hyperinflation (DH) is a consequence of severe airflow obstruction in patients with asthma and chronic obstructive pulmonary disease (COPD). Incorrect setting of positive end-expiratory pressure (PEEP) can lead either to unopposed intrinsic PEEP (iPEEP) (when set too low) or to an increase in lung volume if PEEP is set above iPEEP. DH and iPEEP can lead to haemodynamic compromise [1], increased work of breathing and asynchrony [2]. PEEP setting is challenging because iPEEP is clinically difficult to quantify. Electrical impedance tomography (EIT) provides information on the temporal and spatial heterogeneity of ventilation [3]. EIT may prove useful in optimizing PEEP to overcome gas trapping and DH [4]. We present a method in which the use of EIT allowed selection of PEEP to provide the least DH and inhomogeneity of lung mechanics.

A patient with severe acute COPD exacerbation was on pressure control ventilation: FiO_2_ 0.25, PEEP 10 cmH_2_O, peak pressure 28 cmH_2_O, tidal volume 515 ml, I:E 1:4.4 and set frequency 14/min using an Evita XL ventilator (Draeger–Luebek, Germany). After a short period, the patient developed worsening hypercapnia and clinical evidence of DH. Subsequently a PEEP titration using EIT (Pulmovista®; Draeger–Luebek) was performed to optimize ventilator settings with the aim of minimizing DH.

The patient remained sedated, paralysed and in a supine position throughout the PEEP titration. We measured static iPEEP and compliance using end-inspiratory and end-expiratory hold manoeuvres. iPEEP was measured in a range of set external PEEP, and the iPEEP was calculated as total PEEP minus set PEEP. EIT, tidal volumes, trapped gas volumes and ventilator pressures were then measured at PEEP set to 0 %, 50 %, 80 %, 100 % and 150 % of iPEEP.

EIT waveforms were analysed offline to determine ventilation heterogeneity at different levels of applied PEEP corresponding to 50 %, 80 %, 100 % and 150 % of the static iPEEP. We measured the regional delay of ventilation as a marker of homogeneity of ventilation [5]. We compared this index with oesophageal pressure, with static and dynamic compliance and with arterial blood gases.

EIT allowed determination of the level of PEEP able to achieve greatest lung homogeneity. This level of PEEP was 80 % of iPEEP (Fig. 1). This value also achieved the highest expired tidal volume and the lowest airway resistance. Lung mechanics suggest that PEEP between 80 and 100 % of iPEEP achieves the best compromise between total PEEP and trapped volume (Table 1).

**Fig. 1:**
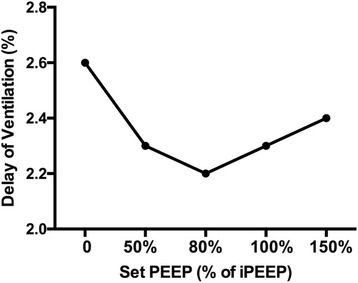
Delays in ventilation as an index of ventilation heterogeneity calculated by EIT using different levels of positive end-expiratory pressure (*PEEP*) as a percentage of the calculated intrinsic PEEP (*iPEEP*). Setting PEEP at 80 % of iPEEP achieves the greatest homogeneity of ventilation

**Table 1:** Measurements of lung mechanics at different PEEP levels

	ZEEP	50 % iPEEP	80 % iPEEP	100 % iPEEP	150 % iPEEP
Set PEEP (cmH_2_O)	0	5	8	10	15
PEEP tot measured (cmH_2_O)	10	10	11	11	15
Plateau pressure (cmH_2_O)	18	21	20	19	21
Expired tidal volume (ml)	505	515	515	500	372
Static compliance (ml/cmH_2_O)	63	47	57	62.5	62
Trapped volume (ml)	390	350	150	80	0

*﻿﻿ZEEP *zero end-expiratory pressure,*﻿ iPEEP* intrinsic positive end-expiratory pressure, *PEEP* positive end-expiratory pressure

This case illustrates how EIT may be useful in assessing regional ventilation and suggesting optimal PEEP. Through optimizing conventional ventilation, bedside EIT may guide ventilatory strategy to reduce hyperinflation, reduce dead space and hence reduce asynchrony and work of breathing without the need for more invasive procedures.

## Abbreviations

COPD: Chronic obstructive pulmonary disease
DH: Dynamic hyperinflation
EIT: Electrical impedance tomography
iPEEP: Intrinsic positive end-expiratory pressure
PEEP: Positive end-expiratory pressure
ZEEP: Zero end-expiratory pressure
